# Effects of COVID-19 Home Confinement on Eating Behaviour and Physical Activity: Results of the ECLB-COVID19 International Online Survey

**DOI:** 10.3390/nu12061583

**Published:** 2020-05-28

**Authors:** Achraf Ammar, Michael Brach, Khaled Trabelsi, Hamdi Chtourou, Omar Boukhris, Liwa Masmoudi, Bassem Bouaziz, Ellen Bentlage, Daniella How, Mona Ahmed, Patrick Müller, Notger Müller, Asma Aloui, Omar Hammouda, Laisa Liane Paineiras-Domingos, Annemarie Braakman-Jansen, Christian Wrede, Sofia Bastoni, Carlos Soares Pernambuco, Leonardo Mataruna, Morteza Taheri, Khadijeh Irandoust, Aïmen Khacharem, Nicola L. Bragazzi, Karim Chamari, Jordan M. Glenn, Nicholas T. Bott, Faiez Gargouri, Lotfi Chaari, Hadj Batatia, Gamal Mohamed Ali, Osama Abdelkarim, Mohamed Jarraya, Kais El Abed, Nizar Souissi, Lisette Van Gemert-Pijnen, Bryan L. Riemann, Laurel Riemann, Wassim Moalla, Jonathan Gómez-Raja, Monique Epstein, Robbert Sanderman, Sebastian V. W. Schulz, Achim Jerg, Ramzi Al-Horani, Taiysir Mansi, Mohamed Jmail, Fernando Barbosa, Fernando Ferreira-Santos, Boštjan Šimunič, Rado Pišot, Andrea Gaggioli, Stephen J. Bailey, Jürgen M. Steinacker, Tarak Driss, Anita Hoekelmann, On Behalf of the ECLB-COVID19 Consortium

**Affiliations:** 1 Institute of Sport Science, Otto-von-Guericke University, 39106 Magdeburg, Germany; anita.hoekelmann@ovgu.de (A.H.);; 2 Research Laboratory, Molecular Bases of Human Pathology, LR12ES17, Faculty of Medicine, University of Sfax, Sfax 3000, Tunisia; omarham007@yahoo.fr; 3 Institute of Sport and Exercise Sciences, University of Münster, 48149 Münster, Germany; michael.brach@uni-muenster.de (M.B.); ellen.bentlage@uni-muenster.de (E.B.); d.how@uni-muenster.de (D.H.); mona.ahmad@uni-muenster.de (M.A.); 4 High Institute of Sport and Physical Education of Sfax, University of Sfax, Sfax 3000, Tunisia; trabelsikhaled@gmail.com (K.T.); liwa.masmoudi@yahoo.fr (L.M.); jarrayam@yahoo.fr (M.J.); kais.elabed@gmail.com (K.E.A.); wassim.moalla@gmail.com (W.M.); 5 Research Laboratory: Education, Motricity, Sport and Health, EM2S, LR19JS01, University of Sfax, Sfax 3000, Tunisia; 6 Research Unit: Physical Activity, Sport, and Health, UR18JS01, National Observatory of Sport, Tunis 1003, Tunisia; h_chtourou@yahoo.fr (H.C.); omarboukhris24@yahoo.com (O.B.); aloui.asma@gmail.com (A.A.); n_souissi@yahoo.fr (N.S.); 7 Higher Institute of Computer Science and Multimedia of Sfax, University of Sfax, Sfax 3000, Tunisia; bassem.bouaziz@isims.usf.tn (B.B.); faiez.gargouri@isims.usf.tn (F.G.); 8 Research Group Neuroprotection, German Center for Neurodegenerative Diseases (DZNE), Leipziger Str. 44, 39120 Magdeburg, Germany; patrick.mueller@dzne.de (P.M.); notger.mueller@dzne.de (N.M.); 9 Medical Faculty, Department of Neurology, Otto-von-Guericke University, Leipziger Str. 44, 39120 Magdeburg, Germany; 10 High Institute of Sport and Physical Education, University of Gafsa, 2112 Gafsa, Tunisia; 11 Laboratório de Vibrações Mecânicas e Práticas Integrativas, LAVIMPI-UERJ, Universidade do Estado do Rio de Janeiro, Rio de Janeiro, RJ 20950-003, Brazil; laisanit@gmail.com; 12 Faculdade Bezerra de Araújo, Rio de Janeiro, Rj 23052-180, Brazil; 13 Department of Psychology, Health & Technology, University of Twente, 7522 Enschede, The Netherland; l.m.a.braakman-jansen@utwente.nl (A.B.-J.); c.wrede@utwente.nl (C.W.); j.vangemert-pijnen@utwente.nl (L.V.G.-P.); 14 Department of Psychology, Università Cattolica del Sacro Cuore, 20123 Milano, Italy; sofia.bastoni2@gmail.com (S.B.); andrea.gaggioli@unicatt.it (A.G.); 15 Laboratório de Fisiologia do Exercício, Estácio de Sá University, Rio de Janeiro 20261-063, Brasil; eremcarlossoares@gmail.com; 16 College of Business Administration, American University in the Emirates, 503000 Dubai Academic City, Dubai, UAE; mataruna@gmail.com; 17 Faculty of social science, Imam Khomeini International University, Qazvin 34148-96818, Iran; taheri_morteza@yahoo.com (M.T.); irandoust@soc.ikiu.ac.ir (K.I.); 18 UVHC, DeVisu, Valenciennes; LIRTES-EA 7313, Université Paris Est Créteil Val de Marne, 94000 Créteil, France; aimen.khacharem@gmail.com; 19 Department of Health Sciences (DISSAL), Postgraduate School of Public Health, University of Genoa, 16132 Genoa, Italy; robertobragazzi@gmail.com; 20 Laboratory for Industrial and Applied Mathematics (LIAM), Department of Mathematics and Statistics, York University, 4700 Keele Street, Toronto, ON M3J 1P3, Canada; 21 ASPETAR, Qatar Orthopaedic and Sports Medicine Hospital, Doha 29222, Qatar; karim.chamari@aspetar.com; 22 Laboratory “Sport Performance Optimization”, (CNMSS); ISSEP Ksar-Said, Manouba University, 2010 Manouba, Tunisia; 23 Exercise Science Research Center, Department of Health, Human Performance and Recreation, University of Arkansas, Fayetteville, AR 72701, USA; jordan@neurotrack.com; 24 Clinical Excellence Research Center, Department of Medicine, Stanford University School of Medicine, Stanford, CA 94305, USA; nbott@stanford.edu; 25 Computer science department, University of Toulouse, IRIT-INP-ENSEEIHT (UMR 5505), BP 7122, 31500 Toulouse, France; chaari.lotfi@gmail.com (L.C.); hadj.batatia@inp-toulouse.fr (H.B.); 26 Faculty of Physical Education, Assiut University, Assiut 71515, Egypt; mdrgamal@yahoo.com (G.M.A.); osamosama@osmail.com (O.A.); 27 Institute for Sports and Sports Science, Karlsruher Institut für Technologie, 76131 Karlsruher, Germany; 28 Department of Health Sciences and Kinesiology, Georgia Southern University, Statesboro, GA 30458, USA; briemann@georgiasouthern.edu; 29 PharmD, BCBS; PharmIAD, Inc, Savannah, GA 30458, USA; pharmiad@comcast.net; 30 FundeSalud, Dept. of Health and Social Services, Government of Extremadura, 06800 Merida, Spain; jonathan.gomez@fundesalud.es; 31 The E-Senior Association, 75020 Paris, France; monique.epstein@gmail.com; 32 Department of Health Psychology, University Medical Center Groningen, University of Groningen, 9712 Groningen, The Netherlands; r.sanderman@umcg.nl; 33 Sports- and Rehabilitation Medicine, Ulm University Hospital, Leimgrubenweg 14, 89075 Ulm, Germany; schulz.sebi@gmx.de (S.V.W.S.); achim.jerg@posteo.de (A.J.); Juergen.Steinacker@uniklinik-ulm.de (J.M.S.); 34 Department of Exercise Science, Yarmouk University, Irbid 21163, Jordan; raalhorani@yu.edu.jo; 35 Faculty of Physical Education, The University of Jordan, 11942 Amman, Jordan; taiysir@hotmail.com; 36 Digital Research Centre of Sfax, Sfax 3000, Tunisia; mohamed.jmaiel@redcad.org; 37 Laboratory of Neuropsychophysiology, Faculty of Psychology and Education Sciences, University of Porto, 4200-135 Porto, Portugal; fernandobarbosa@me.com (F.B.); frsantos@fpce.up.pt (F.F.-S.); 38 Institute for Kinesiology Research, Science and Research Centre Koper, Garibaldijeva 1, 6000 Koper, Slovenia; bostjan.simunic@zrs-kp.si (B.Š.); rado.pisot@zrs-kp.si (R.P.); 39 Applied Technology for Neuro-Psychology Lab, I.R.C.C.S. Istituto Auxologico Italiano, 20149 Milan, Italy; 40 School of Sport, Exercise and Health Sciences, Loughborough University, Loughborough E11 3TU, UK; s.bailey2@lboro.ac.uk; 41 Interdisciplinary Laboratory in Neurosciences, Physiology and Psychology: Physical Activity, Health and Learning (LINP2-2APS), UFR STAPS, UPL, Paris Nanterre University, 92000 Nanterre, France; tarak.driss@parisnanterre.fr

**Keywords:** pandemic, public health, physical activity, nutrition, COVID-19

## Abstract

Background: Public health recommendations and governmental measures during the COVID-19 pandemic have resulted in numerous restrictions on daily living including social distancing, isolation and home confinement. While these measures are imperative to abate the spreading of COVID-19, the impact of these restrictions on health behaviours and lifestyles at home is undefined. Therefore, an international online survey was launched in April 2020, in seven languages, to elucidate the behavioural and lifestyle consequences of COVID-19 restrictions. This report presents the results from the first thousand responders on physical activity (PA) and nutrition behaviours. Methods: Following a structured review of the literature, the “Effects of home Confinement on multiple Lifestyle Behaviours during the COVID-19 outbreak (ECLB-COVID19)” Electronic survey was designed by a steering group of multidisciplinary scientists and academics. The survey was uploaded and shared on the Google online survey platform. Thirty-five research organisations from Europe, North-Africa, Western Asia and the Americas promoted the survey in English, German, French, Arabic, Spanish, Portuguese and Slovenian languages. Questions were presented in a differential format, with questions related to responses “before” and “during” confinement conditions. Results: 1047 replies (54% women) from Asia (36%), Africa (40%), Europe (21%) and other (3%) were included in the analysis. The COVID-19 home confinement had a negative effect on all PA intensity levels (vigorous, moderate, walking and overall). Additionally, daily sitting time increased from 5 to 8 h per day. Food consumption and meal patterns (the type of food, eating out of control, snacks between meals, number of main meals) were more unhealthy during confinement, with only alcohol binge drinking decreasing significantly. Conclusion: While isolation is a necessary measure to protect public health, results indicate that it alters physical activity and eating behaviours in a health compromising direction. A more detailed analysis of survey data will allow for a segregation of these responses in different age groups, countries and other subgroups, which will help develop interventions to mitigate the negative lifestyle behaviours that have manifested during the COVID-19 confinement.

## 1. Introduction

In the face of the present COVID-19 pandemic, public health recommendations and governmental measures have enforced lockdowns and restrictions. While these restrictions help to abate the rate of infection, such limitations result in negative effects by limiting participation in normal daily activities, physical activity (PA), travel and access to many forms of exercise (e.g., closed gyms, no group gatherings, increased social distancing) [1]. Several countries are enforcing curfews, which limit the time to participate in outdoor activities, or are excluding outdoor activities entirely. Such restrictions impose a burden on population health by potentially compromising physical fitness, which is positively associated with the ability to cope with infections and the immunologic and cardiopulmonary complications of more severe outcomes [2,3].

Globally, physical inactivity and poor mental health are among the most important risk factors for major disease morbidity [4]. This holds true not only for the general population, but specifically for older adults and chronically ill patient populations, who are at heightened risk of COVID-19-induced mortality. For children and youth, physical activity is closely coupled to school-related activities, active transport and sport participation [5]. As schools have been closed during the COVID-19 pandemic, this also compromises physical activity participation, therefore increasing the risk of longer-term sedentary behaviours.

In addition to challenges to engage in PA, the closure of food suppliers has placed a burden on normal food-related behaviours [6]. This is noteworthy as good nutrition is important for health and well-being, particularly when the immune system is challenged [7]. Additionally, limited access to fresh food could negatively affect overall physical and mental health [8]. Anxiety and boredom evoked by quarantine are considered risk factors for consuming more food and food of a poorer quality compared to standard living conditions [9]. Combined with the potential for lower levels of PA, impaired nutritional habits could lead to a positive energy balance (i.e., weight gain) [10].

There is limited evidence to evaluate the effect of confinement on PA and dietary behaviours. It is important to investigate how PA and eating behaviours can be affected by lengthy restrictions in order to establish a fundamental basis from which to develop appropriate recommendations for lifestyle modifications during this time. Thus, the main research questions were:

(1) To what extent are physical activity and eating behaviours changed during lengthy restrictions?
(2) Are there changes in mental state, mood and sleep during lengthy restrictions?
(3) Which demographic, cultural and lifestyle factors should be considered as moderators of these effects?

The present paper presents preliminary data on physical activity and nutrition responses before and during home confinement; these data were collected by an international online survey (ECLB-COVID19). Other parts of the survey evaluate general lifestyle [11], socialization [12], mental and mood status [13] and sleep; these findings will be published elsewhere. All papers will share a common method description.

## 2. Materials and Methods

We report findings on the first 1047 replies to an international online survey on mental health and multidimension lifestyle behaviours during home confinement (ECLB-COVID19). ECLB-COVID19 was opened on 1 April 2020, tested by the project’s steering group for a period of one week and spread worldwide on 6 April 2020. Thirty-five research organizations from Europe, North-Africa, Western Asia and the Americas promoted dissemination and administration of the survey. ECLB-COVID19 was administered in English, German, French, Arabic, Spanish, Portuguese and Slovenian languages (other languages including Dutch, Persian, Italian, Greek, Russian, Indian and Malayalam have since been added). The survey included 64 questions on health, mental well-being, mood, life satisfaction and multidimension lifestyle behaviours (physical activity, diet, social participation, sleep, technology use, need of psychosocial support). All questions were presented in a differential format, to be answered directly in sequence regarding “before” and “during” confinement conditions [11,12,13,14]. The study was conducted according to the Declaration of Helsinki. The protocol and the consent form were fully approved (identification code: 62/20) by the Otto von Guericke University Ethics Committee.

### 2.1. Survey Development and Promotion

The ECLB-COVID19 electronic survey was designed by a steering group of multidisciplinary scientists and academics (i.e., human science, sport science, neuropsychology and computer science) at the University of Magdeburg (principal investigator), the University of Sfax, the University of Münster and the University of Paris-Nanterre, following a structured review of the literature. The survey was then reviewed and edited by over 50 colleagues and experts worldwide. The survey was uploaded and shared on the Google online survey platform. A link to the electronic survey was distributed worldwide by consortium colleagues via a range of methods: invitation via e-mails, shared in consortium’s faculties official pages, ResearchGate™, LinkedIn™, Facebook™, WhatsApp™ and Twitter™. The general public were also involved in the dissemination plans through the promotion of the ECLB-COVID19 survey in their personal networks. The survey included an introductory page describing the background and the aims of the survey, the consortium, ethics information for participants and the option to choose one of seven languages available at the time (English, German, French, Arabic, Spanish, Portuguese and Slovenian) [11,12,13,14]. The present study focuses on the first thousand responses (i.e., 1047 participants), which were reached on 11 April 2020, approximately one week after survey dissemination. This survey was open for all people worldwide aged 18 years or older. People with cognitive decline were excluded.

### 2.2. Data Privacy and Consent of Participation

During the informed consent process, survey participants were assured all data would be used only for research purposes. Participants’ answers are anonymous and confidential according to Google’s privacy policy (https://policies.google.com/privacy?hl=en). Participants were not permitted to provide their names or contact information. Additionally, participants were able to stop study participation and leave the questionnaire at any stage before the submission process; if doing so, their responses would not be saved. Responses were saved only by clicking on the provided “submit” button. By completing the survey, participants acknowledged their voluntary consent to participate in this anonymous study. Participants were requested to be honest in their responses [11,12,13,14].

### 2.3. Survey Questionnaires

The ECLB-COVID19 is a multicountry electronic survey designed to assess change in multiple lifestyle behaviours during the COVID-19 outbreak. Therefore, a collection of validated and/or crisis-oriented brief questionnaires were included [11,12,13,14]. These questionnaires assess mental well-being (Short Warwick-Edinburgh Mental Well-being Scale (SWEMWBS) [11,13,15]), mood and feeling (Short Mood and Feelings Questionnaire (SMFQ) [11,13,16]), life satisfaction (Short Life Satisfaction Questionnaire for Lockdowns (SLSQL) [11,12]), social participation (Short Social Participation Questionnaire for Lockdowns (SSPQL) [11,12]), physical activity (International Physical Activity Questionnaire Short Form (IPAQ-SF) [11,14,17,18]), diet behaviours (Short Diet Behaviours Questionnaire for Lockdowns (SDBQL) [11,14]), sleep quality (Pittsburgh Sleep Quality Index (PSQI) [19]) and some key questions assessing the technology-use behaviours (Short Technology-use Behaviours Questionnaire for Lockdowns (STBQL) [11]), demographic information and the need of psychosocial support [11]. Reliability of the shortened and/or newly adopted questionnaires was tested by the project steering group through piloting, prior to survey administration. These brief crisis-oriented questionnaires demonstrated good to excellent test–retest reliability coefficients (r = 0.84–0.96). A multilanguage validated version already existed for the majority of these questionnaires and/or questions. However, for questionnaires that did not already exist in multilanguage versions, we followed the procedure of translation and back-translation, with an additional review for all language versions from the international scientists of our consortium. As a result, a total number of 64 items were included in the ECLB-COVID19 online survey in a differential format (i.e., each item or question requested two answers, one regarding the period before and the other regarding the period during confinement). The participants were guided to compare the situations [11,12,13,14]. Given the large number of questions included, the present paper focuses on the IPAQ-SF and the newly developed SDBQL as brief crisis-oriented tools.

#### 2.3.1. International Physical Activity Questionnaire Short Form (IPAQ-SF)

According to the official IPAQ-SF guidelines, data from the IPAQ-SF are summed within each item (i.e., vigorous intensity, moderate intensity and walking) to estimate the total amount of time spent engaged in PA per week [17,18]. Total weekly PA (MET-min·week-1) was estimated by adding the products of reported time for each item by a MET value that was specific to each category of PA. We assigned two different sets of MET values. The first set was the original values (original IPAQ) based on the official IPAQ guidelines for young and middle-aged adult (18–65 years old): vigorous PA = 8.0 METs, moderate PA = 4.0 METs and walking = 3.3 METs. The other set used modified values (modified IPAQ), which we had devised for use with elderly adults (>65 years old), as reported by Stewart et al. [20] and Yasunaga et al. [21]: vigorous PA = 5.3 METs, moderate PA = 3.0 METs and walking = 2.5 METs. Additionally, we added the total PA (sum of performed vigorous, moderate and walking activity) as a fourth item and sitting time as fifth item.

#### 2.3.2. Short Diet Behaviour Questionnaire for Lockdowns

The SDBQ-L is a newly developed crisis-oriented short questionnaire to assess dietary behaviour before and during the lockdown period. The SDBQ-L has five questions related to “1. unhealthy food”, “2. eating out of control”, “3. snacks between meals”, “4. alcohol binge drinking”, and “5. number of main meals/day” in parts referring to the Nutricalc questionnaire, Swiss Society of Nutrition [22]. Regarding the first question related to unhealthy food, explanation was provided with the question as follows: “1. How likely are you to have an unhealthy diet/food? (high in calories from sugar or fat, colorants, salt and tropical oils; and low in fibre and vitamins (e.g., fried potato crisps/chips, cakes, white sauces)”. The response choices and their designated scores were as follows: “Never” = 0; “Sometimes” = 1; “Most of the time” = 2; “Always” = 3. These choices and points were applied for the first four questions. The choices and the designated scores for the fifth question were as follows: “1–2” = 1; “3” = 0; “4” = 1; “5” = 2; “>5” = 3. A recent study by Harder-Lauridsen et al. [23] showed parallel increases in the incidence of obesity and diabetes with increases in the number of daily meals. Consequently, it is suggested that increasing number of meals and/or snacks between main meals is considered unhealthy diet behaviour. Lower scores (0 to 1) in these five SDBQ-L questions indicate participants are less likely to (i) have unhealthy food, (ii) eat out of control, (iii) have high number of snacks between meals, (iv) drink alcohol out of control and (v) have high number of meals. However, higher scores (2 to 3) in these questions indicate participants are more likely to engage in these aforementioned unhealthy diet habits.

The total score of this questionnaire corresponded to the sum of the scores in the five questions. The total score for the SDBQ-L is from “0” to “15”, where “0” designates no unhealthy dietary behaviours and “15” designates severe unhealthy dietary behaviours.

### 2.4. Data Analysis

Descriptive statistics were used to define the proportion of responses for each question and the total distribution in the total score of each questionnaire. All statistical analyses were performed using the commercial statistical software STATISTICA (StatSoft, Paris, France, version 10.0) and Microsoft Excel 2010. Normality of the data distribution was confirmed using the Shapiro–Wilks W-test. Values were computed and reported as mean ± SD (standard deviation). To assess for significant differences in responses before and during the confinement period, paired samples *t*-tests were used. Effect size (Cohen’s d) was calculated to determine the magnitude of the change of the score and was interpreted using the following criteria: 0.2 (small), 0.5 (moderate) and 0.8 (large) [24]. Statistical significance was accepted as *p* < 0.05.

## 3. Results

### 3.1. Sample Description

The present study focused on the first thousand responses (i.e., 1047 participants), which were reached on 11 April 2020. Overall, 54% of the participants were female and the participants were from Asian (36%, mostly from Western Asia), African (40%, mostly from North Africa), European (21%) and other (3%) countries. Age, health status, employment status, level of education and marital status are presented in Table 1.

### 3.2. Physical Activity before and during the Confinement Period

Responses to the physical activity questionnaire recorded before and during home confinement are presented in Table 2.

#### 3.2.1. Vigorous Intensity

The number of days/week and minutes/day of vigorous intensity PA during, compared to before, home confinement decreased by 22.7% (*t* = 7.75, *p* < 0.001, *d* = 0.374) and 33.1% (*t* = 9.75 *p* < 0.001, *d* = 0.542), respectively. Additionally, MET values of vigorous intensity PA were 36.9% lower during, compared to before, home confinement (*t* = 6.68, *p* < 0.001, *d* = 0.315).

#### 3.2.2. Moderate Intensity

The number of days/week of moderate intensity PA decreased by 24% during home confinement (*t* = 7.89, *p* < 0.001, *d* = 0.396). Likewise, the number of minutes/day of moderate intensity PA decreased by 33.4% during home confinement (*t* = 7.85, *p* < 0.001, *d* = 0.343). Additionally, MET values of moderate intensity were 34.7% lower during home confinement (*t* = 5.24, *p* < 0.001, *d* = 0.204).

#### 3.2.3. Walking

The number of days/week of walking decreased by 35% during home confinement (*t* = 15.80, *p* < 0.001, *d* = 0.677). Likewise, the number of minutes/day of walking decreased by 34% during home confinement (*t* = 9.34, *p* < 0.001, *d* = 0.389). Additionally, MET values of walking were 42.7% lower during home confinement (*t* = 9.03, *p* < 0.001, *d* = 0.361).

#### 3.2.4. All Physical Activity (PA)

The number of days/week of all PA decreased by 24% during home confinement (*t* = 15.61, *p* < 0.001, *d* = 0.482). Likewise, the number of minutes/day of all PA decreased by 33.5% during home confinement (*t* = 12.51, *p* < 0.001, *d* = 0.387). Additionally, MET values of all PA were 38% lower during home confinement (*t* = 9.14, *p* < 0.001, *d* = 0.283).

#### 3.2.5. Sitting

Statistical analysis reported that the number of hours/day of sitting increased by 28.6% during home confinement (*t* = −25.61, *p* < 0.001, *d* = 1.130).

### 3.3. Dietary Behaviours before and during the Confinement Period

**Figure 1:**
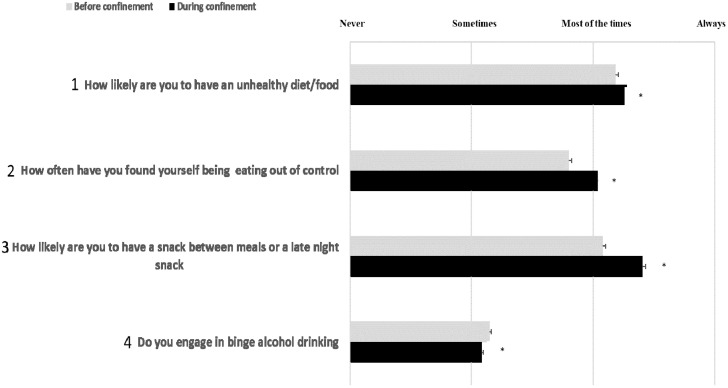
Participants’ scores in response to the related diet behaviour questions. *: Significant differences between “before” and “during” COVID-19 home confinement period.

Recorded scores in responses to the diet behaviour questionnaire before and during home confinement are presented in Figure 1 (question 1 to 4) and 2 (question 5). For detailed distribution of responses (in %), please see Table S1 (Supplementary File).

#### 3.3.1. Total Score of Diet

Statistical analysis reported that the total score of diet was 4.4% higher during, compared to before, home confinement (*t* = −10.66, *p* < 0.001, *d* = 0.50).

#### 3.3.2. Question 1 (Q1): Unhealthy Food

The score of Q1 (consuming unhealthy food; Figure 1) was significantly higher during home confinement (*t* = −3.46, *p* < 0.001, *d* = 0.14). The percentage of responses that indicated consuming unhealthy food either most of the time or always was higher during home confinement (23.3% vs. 18.4% for most of the time and 10.9% vs. 6.2% for always).

#### 3.3.3. Question 2 (Q2): Eating out of Control

The score of Q2 (eating out of control; Figure 1) was significantly higher during home confinement (*t* = −9.44, *p* < 0.001, *d* = 0.22). The percentage of responses that indicated eating out of control either most of the time or always was higher during home confinement (20.4% vs. 9.7% for most of the time and 9.6% vs. 2.3% for always).

#### 3.3.4. Question 3 (Q3): Snacking between Meals

The number of snacks between meals or late-night snacking estimated by Q3 (Figure 1) increased significantly during home confinement (*t* = −6.89, *p* < 0.001, *d* = 0.30). The percentage of responses that indicated having a snack between meals or late-night snack either most of the time or always was higher during home confinement (24.4% vs. 13.9% for most of the time and 15.4% vs. 6.4% for always).

#### 3.3.5. Question 4 (Q4): Alcohol Binge Drinking

The score of Q4 (binge alcohol drink; Figure 1) decreased significantly during home confinement (*t* = −12.16, *p* < 0.001, *d* = 0.58). In fact, the percentage of responses that indicated alcohol binge drinking either sometimes, most of the time or always was lower during home confinement (5.4% vs. 10.1% for sometimes, 1.2% vs. 1.8% for most of the time and 0.2% vs. 0.4% for always).

#### 3.3.6. Question 5 (Q5): Number of Main Meals/Day

**Figure 2:**
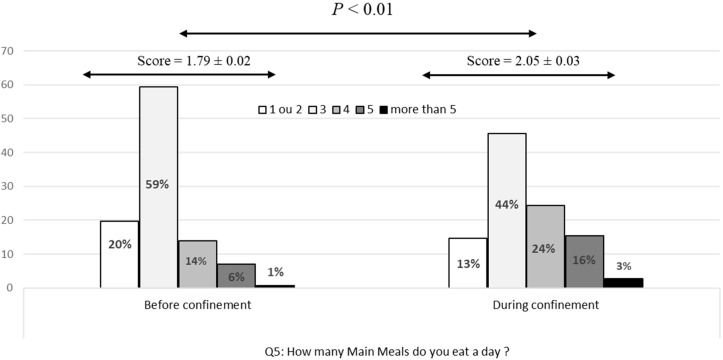
Response distribution (in %) and recorded score according to the number of main meals per day before and during confinement.

The number of main meals estimated by Q5 (Figure 2) was significantly higher during home confinement (*t* = −5.83, *p* < 0.001, *d* = 0.22). The percentages of responses that indicated 4, 5 and more than 5 main meals were higher during home confinement (14.5% vs. 6.6% for 4 main meals, 6.3% vs. 2.4% for 5 main meals and 2.8% vs. 0.8% for more than 5 main meals).

## 4. Discussion

This report presents the preliminary data from an online survey collected in eight languages, comparing physical activity (PA) and dietary behaviours before and during home confinement as a result of COVID-19. There were 1047 replies (54% women) from Western Asia (36%), North Africa (40%), Europe (21%) and other countries (3%), which revealed that the COVID-19 home confinement has had a negative effect on all levels of PA (vigorous, moderate, walking and overall) and an increase in daily sitting time by more than 28%. Additionally, an unhealthy pattern of food consumption (the type of food, eating out of control, snacks between meals and number of main meals) was exhibited. Only alcohol binge drinking decreased significantly.

Despite recommendations that home confinement should not hinder people from being physically active [25], present results show that there has been a decline in all PA levels during the COVID-19 home confinement period. While the effect size is small to medium for most parameters, the 35% reduction in number of days per week walking is medium to large. In fact, 2.45 days is a serious change, independent from the number of walking days before confinement. However, the most prominent change was in sitting behaviour, which increased more than a full standard deviation (very large effect size: *d* = 1.13), most likely due to the increased time that people were required to stay within their quarantine location. Indeed, 29% of the sample reported sitting for 6–8 h a day during confinement (vs. 24% before), a threshold area which Patterson et al. [26] suggested causes an increase in disease and mortality risks. Far more serious was the proportion of individuals who sat for more than 8 h a day, which increased from 16% to 40% during confinement. Preliminary data indicate that 41% of the sample increased their sitting behaviour by only 1 h or less, but this increased by five hours or more for 27% of the sample.

The results of this survey concur with recent studies demonstrating that the current COVID-19 home confinement could dramatically impact lifestyle activities globally, including participation in sports and PA engagement [12,27]. The restrictions have reduced overall PA (number of days and number of hours) and access to exercise. In spite of an increased offering of PA guidance and classes available on social media, present results indicate that it has not been possible for individuals to adequately maintain their normal PA patterns with home activities. The decline in PA was accompanied by increased sedentary (sitting) behaviour. However, the extent to which PA participation is impacted by the current COVID-19 pandemic will be linked to the stringency of individual government confinement policies. It is already shown in China that different regional policies and socio-economic factors were associated with differences in PA [1]. These factors need to be considered when designing and promoting PA interventions for the COVID-19 pandemic. It was recently demonstrated that individuals demonstrate a greater use (15%) of Information and Communications Technology (ICT) during the confinement period [11]. Therefore, future PA intervention to foster an Active and Healthy Confinement Lifestyle (AHCL) during pandemic can be based on ICT solutions, such as home-based exergames and fitness apps.

The results of this survey also found that, in contrast to the guidance of the World Health Organization [6,7], people changed their eating behaviours, with increased consumption of unhealthy food, eating out of control, more snacking between meals and an overall higher number of main meals [28]. Regarding dietary behaviours, there seems to be no single behavioural problem. While a medium effect size (*d* = 0.5) was recorded for total score, a small effect size was registered for the type of food, number of main meals and eating out of control. The effect size for snacks between meals was medium. Binge drinking of alcohol showed the largest effect size (*d* = 0.58) but, conversely, in a healthy direction. This may be due to the fact that younger individuals are less likely to be surrounded by other drinking peers [29].

The negative changes in the majority of eating behaviours could be attributed to eating out of anxiety or boredom [9], a dip in motivation to participate in PA or maintain healthy eating [30] or an increase in mood-driven eating [9]. Alternative support for motivation during home confinement may be sourced from assistive technologies such as apps, streaming services and social media. In order to counteract poor dietary behaviours, meal planning and controlling food composition and meals’ caloric content using ICT-based solutions such as *m*health and nutrition apps may be the best approach to combating unhealthy eating habits while in confinement [9,11].

The results reported in this report should be utilised for further research and development in public health promotion during the COVID-19 pandemic. Motivating people to stand up can be a first step of health promotion against sedentary behaviour. It was recently suggested that in times of restrictions due to the COVID19 pandemic, breaking up prolonged sitting with simple measures, such as alternating between sitting and standing for 30 min periods, may result in meaningful increases in energy expenditure, thus promoting metabolic health in terms of glycaemic control for both healthy and diseased individuals [31,32]. Individuals may have improved metabolism and other health outcomes during the COVID-19 home confinement by adhering to the following dietary behaviours: (i) reducing meal frequency, (ii) consuming regular (i.e., breakfast (about 40% of daily total energy), (iii) lunch (30% of daily total energy) and (iv) dinner (30% of daily total energy)) and (v) good quality meals (e.g., more fresh vegetables, good quality protein source, avoiding refined and high glycaemic foods), and (vi) adapting intermittent or a long fasting period (i.e., more than 12 h) [7,31]. Further research should address (i) insight into subpopulations for the development of interventions to address their needs, (ii) interference of diet and PA behaviours, for improving interventions, and (iii) identification of conditions for successfully maintaining a healthy lifestyle before as well as during isolation.

Although many ideas and recommendations already exist [7,27,31,32,33], individuals seem to need more support to effectively use the services offered and to understand the consequences of inaction. Technology and social media allow for innovative health behaviour support via fitness applications and video streaming and motivation and gamification support; adding the beneficial social aspect is also very important to encourage maintenance of physical activity behaviours.

## 5. Strengths and Limitations

The strengths of this research project include a survey provided in multiple languages, which has been widely distributed in several continents. Scientists from different disciplines and many countries cooperated to make this possible. However, there were also limitations of the low-threshold strategy in that it did not allow for narrow target groups with defined inclusion and exclusion criteria. Thus, a collection from a representative sample cannot be expected. Only during post hoc studies can criteria-based subsamples be analysed. The validity of answers is a general problem of online surveys and we attempted to address this by the differential approach described in the methods section.

## 6. Conclusions and Future Perspectives

The preliminary results of the survey indicate a negative effect of home confinement on PA and diet behaviour with a significant increase in sitting time and unhealthy diet, indicative of a more sedentary lifestyle. These observations have potential implications that could aid the development of PA and nutritional recommendations to maintain health during the COVID-19 pandemic. The major perspectives of the ECLB-COVID 19 multicentre study are to target more affected countries and to collect more responses, allowing a between-country comparison and also separate analysis of each country’s data. Indeed, identifying exact behavioural changes in each country will provide better-informed decisions during the reopening process.

## Figures and Tables

**Table 1:** Demographic characteristics of the participants.

Variables		*n*	(%)
Gender			
	Male	484	(46.2%)
	Female	563	(53.8%)
Continent			
	North Africa	419	(40%)
	Western Asia	377	(36%)
	Europe	220	(21%)
	Other	31	(3%)
Age (years)			
	18–35	577	(55.1%)
	36–55	367	(35.1%)
	>55	103	(9.8%)
Level of education			
	Master/doctorate degree	527	(50.3%)
	Bachelor’s degree	397	(37.9%)
	Professional degree	28	(2.7%)
	High school graduate, diploma or the equivalent	69	(6.6%)
	No schooling completed	26	(2.5%)
Marital status			
	Single	455	(43.5%)
	Married/Living as couple	562	(53.7%)
	Widowed/Divorced/Separated	30	(2.9%)
Employment status			
	Employed for wages	538	(51.4%)
	Self-employed	74	(7.1%)
	Out of work/Unemployed	75	(7.2%)
	A student	259	(24.7%)
	Retired	23	(2.2%)
	Unable to work	9	(0.9%)
	Problem caused by COVID-19	59	(5.6%)
	Other	10	(1%)
Health state			
	Healthy	956	(91.3%)
	With risk factors for cardiovascular disease	81	(7.7%)
	With cardiovascular disease	10	(1%)

**Table 2:** Responses to the physical activity questionnaire recorded before and during home confinement.

		before Confinement	during Confinement	Δ (Δ%)	*t* Test	*p* Value	Cohen’s d
Vigorous intensity	Days/week	1.97 ± 2.11	1.52 ± 2.03	0.45 (22.7%)	7.751	<0.001	0.374
	min/week	38.7 ± 58.1	26.0 ± 47.8	12.83 (33.1%)	9.759	<0.001	0.542
	MET values	1168 ± 2468.7	737.2 ± 1844.5	430.8 (36.9%)	6.683	<0.001	0.315
Moderate intensity	Days/week	1.79 ± 2.08	1.36 ± 1.95	0.43 (24.0%)	7.898	<0.001	0.396
	min/week	32.1 ± 49	21.4 ± 37.3	10.72 (33.4%)	7.858	<0.001	0.343
	MET values	446.4 ± 920.2	291.5 ± 772.7	154.8 (34.7%)	5.247	<0.001	0.204
Walking	Days/week	3.59 ± 2.58	2.33 ± 2.48	1.26 (35.0%)	15.806	<0.001	0.677
	min/week	37.2 ± 46.8	24.6 ± 34.1	12.64 (34.0%)	9.343	<0.001	0.389
	MET values	578.3 ± 917.1	331.4 ± 640.2	246.8 (42.7%)	9.039	<0.001	0.361
All PA	Days/week	5.04 ± 2.51	3.83 ± 2.82	1.21 (24.0%)	15.611	<0.001	0.482
	min/week	108 ± 114.2	71.8 ± 88.2	36.19 (33.5%)	12.510	<0.001	0.387
	MET values	2192.6 ± 3300.7	1360.2 ± 2545.2	832.5 (38.0%)	9.146	<0.001	0.283
Sitting	Hours/day	5.31 ± 3.65	8.41 ± 5.11	3.10 (28.6%)	−25.030	<0.001	1.130

## Supplementary Materials

The following are available online at https://www.mdpi.com/2072-6643/12/6/1583/s1, Table S1: Distribution of responses (%) in each item of the diet behaviour questionnaire

## References

[1] Hossain M.M., Sultana A., Purohit N. (2020). Mental health outcomes of quarantine and isolation for infection prevention: A systematic umbrella review of the global evidence. SSRN Electron. J..

[2] Bloch W., Halle M., Steinacker J.M. (2020). Sport in times of Corona (Sport in Zeiten von Corona). Ger. J. Sports Med..

[3] Steinacker J.M., Bloch W., Halle M., Mayer F., Meyer T., Hirschmüller A., Roecker K., Wolfarth B., Nieß A., Reinsberger C. (2020). Merkblatt: Gesundheitssituation für Sportler durch die aktuelle Coronavirus-Pandemie (SARS-CoV-2/COVID-19). Dtsch. Z. Sportmed..

[4] Hallal P.C., Andersen L.B., Bull F.C., Guthold R., Haskell W., Ekelund U., Lancet Physical Activity Series Working Group (2012). Global physical activity levels: Surveillance progress, pitfalls, and prospects. Lancet.

[5] Hoffmann B., Kobel S., Wartha O., Kettner S., Dreyhaupt J., Steinacker J.M. (2019). High sedentary time in children is not only due to screen media use: A cross-sectional study. BMC Pediatr..

[6] WHO (2020). Food and Nutrition during Self-Quarantine: What to Choose and How to Eat Healthy.

[7] WHO. http://www.euro.who.int/en/health-topics/health-emergencies/coronavirus-covid-19/novel-coronavirus-2019-ncov-technical-guidance/food-and-nutrition-tips-during-self-quarantine.

[8] WHO (2020). Be Active during COVID-19.

[9] BDA Eating Well during Coronavirus/COVID-19. https://www.bda.uk.com/resource/eating-well-during-coronavirus-covid-19.html.

[10] Hill J.O., Wyatt H.R., Peters J.C. (2012). Energy balance and obesity. Circulation.

[11] Ammar A., Trabelsi K., Brach M., Chtourou H., Boukhris O., Masmoudi L., Bouaziz B., Bentlage E., How D., Ahmed M. (2020). Effects of home confinement on mental health and lifestyle behaviours during the COVID-19 outbreak: Insight from the “ECLB-COVID19” multi countries survey. medRxiv.

[12] Ammar A., Brach M., Trabelsi K., Chtourou H., Boukhris O., Masmoudi L., Bouaziz B., Bentlage E., How D., Ahmed M. (2020). Effects of COVID-19 home confinement on social participation and life satisfaction: Preliminary results of the ECLB-COVID19 international online-survey. medRxiv.

[13] Ammar A., Mueller P., Trabelsi K., Chtourou H., Boukhris O., Masmoudi L., Bouaziz B., Brach M., Schmicker M., Bentlage E. (2020). Emotional consequences of COVID-19 home confinement: The ECLB-COVID19 multicenter study. medRxiv.

[14] Ammar A., Brach M., Trabelsi K., Chtourou H., Boukhris O., Masmoudi L., Bouaziz B., Bentlage E., How D., Ahmed M. (2020). Effects of COVID-19 home confinement on physical activity and eating behaviour Preliminary results of the ECLB-COVID19 international online-survey. medRxiv.

[15] Ng Fat L., Scholes S., Boniface S., Mindell J., Stewart-Brown S. (2017). Evaluating and establishing the national norms for mental well-being using the short Warwick-Edinburgh Mental Well-being Scale (SWEMWBS): Findings from the Health Survey for England. Qual. Life Res..

[16] Thabrew H., Stasiak K., Bavin L.M., Frampton C., Merry S. (2018). Validation of the Mood and Feelings Questionnaire (MFQ) and Short Mood and Feelings Questionnaire (SMFQ) in New Zealand help-seeking adolescents. Int. J. Meth. Psychiatr. Res..

[17] Graig C.L., Marshall A.L., Sjöström M., Bauman A.E., Booth M.L., Ainsworth B.E., Pratt M., Ekelund U.L.F., Yngve A., Sallis J.F. (2003). International Physical Activity Questionnaire: 12-Contry Reliability and Validity. Med. Sci. Sports Exerc..

[18] Lee P.H., Macfarlane D.J., Lam T.H., Stewart S.M. (2011). Validity of the international physical activity questionnaire short from (PAQ-SF): A systematic review. Int. J. Behav. Nutr. Phys. Act..

[19] Buysse D.J., Reynolds C.F., Monk T.H., Berman S.R., Kupfer D.J. (1989). The Pittsburgh Sleep Quality Index: A new instrument for psychiatric practice and research. Psychiatry Res..

[20] Stewart A.L., Mills K.M., King A.C., Haskell W.L., Gillis D.A.W.N., Ritter P.L. (2001). CHAMPS physical activity questionnaire for older adults: Outcomes for interventions. Med. Sci. Sports Exerc..

[21] Yasunaga A., Park H., Watanabe E., Togo F., Park S., Shephard R.J., Aoyagi Y. (2007). Development and evaluation of the physical activity questionnaire for elderly Japanese: The Nakanojo study. J. Aging Phys. Act..

[22] Nutricalc Questionnaire, Swiss Society of Nutrition. https://journals.plos.org/plosone/article/file?type=supplementary&id=info:doi/10.1371/journal.pone.0143293.s003.

[23] Harder-Lauridsen N.M., Rosenberg A., Benatti F.B., Damm J.A., Thomsen C., Mortensen E.L., Pedersen B.K., Krogh-Madsen R. (2017). Ramadan model of intermittent fasting for 28 d had no major effect on body composition, glucose metabolism, or cognitive functions in healthy lean men. Nutrition.

[24] Cohen J. (1988). Statistical Power Analysis for the Behavioral Sciences.

[25] WHO Physical Activity and Adults. Recommended Levels of Physical Activity for Adults Aged 18–64 Years.

[26] Patterson R., McNamara E., Tainio M., de Sá T.H., Smith A.D., Sharp S.J., Edwards P., Woodcock J., Brage S., Wijndaele K. (2018). Sedentary behaviour and risk of all-cause, cardiovascular and cancer mortality, and incident type 2 diabetes: A systematic review and dose response meta-analysis. Eur. J. Epidemiol..

[27] Oliveira Neto L., Elsangedy H.M., Tavares V.D.O., Teixeira C.V.L.S., Behm D.G., Da Silva-Grigoletto M.E. (2020). #TrainingInHome—Training at home during the COVID-19 (SARS-COV2) pandemic: Physical exercise and behavior-based approach. Rev. Bras. Fisiol. Exerc..

[28] NNEDPro (2020). Combatting COVID-19: A 10-Point Summary on Diet, Nutrition and the Role of Micronutrients. https://www.nnedpro.org.uk/post/combatting-covid-19.

[29] Richardson B., Fuller-Tyszkiewicz M., Liknaitzky P., Arulkadacham L., Dvorak R., Staiger P.K. (2019). Ecological momentary assessment of drinking in young adults: An investigation into social context, affect and motives. Addict. Behav..

[30] Gardner B., Rebar A.L. Habit Formation and Behavior Change. https://uni-muenster.sciebo.de/apps/files/?dir=/2020_PROCare4Life_3100050300/02_Online%20survey%20ECLB-COVID19&fileid=1748656124#pdfviewer.

[31] Narici M., De Vito G., Franchi M., Paoli A., Moro T., Marcolin G., Grassi B., Baldassarre G., Zuccarelli L., Biolo G. (2020). Impact of sedentarism due to the COVID-19 home confinement on neuromuscular, cardiovascular and metabolic health: Physiological and pathophysiological implications and recommendations for physical and nutritional countermeasures. Eur. J. Sport Sci..

[32] Joy L. (2020). Staying Active during COVID-19. https://www.exerciseismedicine.org/support_page.php/stories/?b=892.

[33] Chaari L., Golubnichaya O. (2020). Covid-19 pandemic by the “real-time” monitoring: The Tunisian case and lessons for global epidemics in the context of 3PM strategies. EPMA J..

