# Supplementary material for: Effects of COVID-19 Home Confinement on Eating Behaviour and Physical Activity: Results of the ECLB-COVID19 International Online Survey
Source: Nutrients. 2020 May 28;12(6):1583. doi: 10.3390/nu12061583 (PMC7352706; doi:10.3390/nu12061583)
Supplement: Supplementary file 1 [file nutrients-12-01583-s001.pdf]

Supplementary Material

Table S1: Distribution of responses (%) in each item of the diet behavior questionnaire

**Question/responses**				**% of responses (Q1-Q4)**				**% of responses (Q5)**				
**Question number**	Text	Period	Mean ± SD	Never	Sometimes	Most of the time	Always	1 ou 2	3	4	5	more than 5
**Q1**	How likely are you to have an unhealthy diet/food	Before	2.18±0.02	12.23	63.13	18.43	6,21	N/A				
		During	2.27±0.03	17.38	48.42	23.30	10,89					
**Q2**	How often have you found yourself being eating out of control	Before	1.80±0.02	34.19	53.77	9.74	2,29					
		During	2.04±0.03	35.82	34.19	20.44	9,55					
**Q3**	How likely are you to have a snack between meals or a late-night snack	Before	2.08±0.02	19.77	59.41	13.85	6,97					
		During	2.40±0.03	14.71	45.56	24.36	15,38					
**Q4**	Do you engage in binge alcohol drinking	Before	1.15±0.01	87.68	10.12	1.81	0,38					
		During	1.08±0.01	93.22	5.35	1.24	0,19					
**Q5**	How many main meals do you eat a day	Before	1.79±0.02	N/A				35.15	55.11	6.59	2.39	0.76
		During	2.05±0.03					29.99	46.42	14.52	6.30	2.77
